# *Eucommia ulmoides* Bark Extract Attenuates Oxidative Damage and H_2_O_2_-Induced Hair Follicle Suppression in Cellular and Mouse Models

**DOI:** 10.3390/molecules31162863

**Published:** 2026-08-17

**Authors:** Xiaojin Liu, Shuyue Chen, Yanyan Zhang, Yaqian Qiu, Yefan Gu

**Affiliations:** 1 Anhui Provincial Key Laboratory of Molecular Enzymology and Mechanism of Major Metabolic Diseases, College of Life Sciences, Anhui Normal University, Wuhu 241000, China; 2 Anhui Provincial Engineering Research Centre for Molecular Detection and Diagnostics, College of Life Sciences, Anhui Normal University, Wuhu 241000, China

**Keywords:** *Eucommia ulmoides*, HPLC-DAD, geniposidic acid, chlorogenic acid, oxidative stress, Nrf2, HO-1, dermal papilla cells, B16 melanoma cells, oxidative stress-induced hair follicle suppression, 5α-reductase inhibition

## Abstract

Oxidative stress contributes to hair follicle aging, melanocyte dysfunction, and androgen-related hair disorders, yet chemically characterized botanical interventions with multi-target activities remain incompletely defined. This study evaluated the antioxidant, follicle-supporting, and antiandrogenic potential of *Eucommia ulmoides* bark extract (EUE) using integrated in vitro and in vivo approaches and characterized EUE by HPLC-DAD using geniposidic acid and chlorogenic acid as representative marker compounds. Chlorogenic acid and geniposidic acid were present at 3.21 ± 0.01 and 1.88 ± 0.15 mg/g extract, respectively. In replicate-well assays using H_2_O_2_-challenged B16 melanoma cells, EUE increased superoxide dismutase activity and reduced malondialdehyde accumulation. Technical-triplicate RT-qPCR measurements and a single Western blot experiment showed descriptive changes in Nrf2 and HO-1 markers; these observations are exploratory and require independent biological replication. In HDPCs, replicate-well viability assays and technical-triplicate RT-qPCR measurements indicated concentration-associated viability changes and descriptive changes in VEGF, Wnt5a, and β-catenin transcripts. EUE also inhibited 5α-reductase activity in a cell-free assay, although the non-monotonic response and the much higher mass concentrations used relative to finasteride preclude potency equivalence. In male C57BL/6 mice, topical EUE attenuated H_2_O_2_-induced suppression of hair follicle elongation and improved follicular morphology. Overall, EUE is a chemically characterized botanical extract with preliminary biological activities relevant to oxidative stress- and androgen-associated hair disorders; independent replication, active-compound identification, marker-specific permeation, and protein-level validation of the measured markers remain necessary.

## 1. Introduction

Hair loss and premature graying are highly prevalent dermatological conditions influenced by intrinsic aging, environmental stressors, and genetic predisposition. Accumulating evidence highlights oxidative stress as a central pathological driver in follicular aging, melanocyte degeneration, and hair miniaturization. Excessive reactive oxygen species (ROS), such as H_2_O_2_, disrupt redox homeostasis, impair mitochondrial function, damage cellular macromolecules, and ultimately trigger senescence or apoptosis in follicular keratinocytes and melanocytes [1,2,3].

Maintenance of hair follicle integrity relies on coordinated interactions among redox regulation, stem cell activation, angiogenesis, and hormonal metabolism. The Nrf2/HO-1 axis is an important antioxidative defense system that supports cellular viability under oxidative challenge [4,5]. Wnt/β-catenin-associated signaling is involved in follicular morphogenesis, stem cell activation, and melanocyte differentiation [6,7], while VEGF-mediated angiogenesis contributes to nutrient supply during active follicular growth [8]. Conversely, androgen metabolism, particularly DHT formation via 5α-reductase, contributes to follicular miniaturization in androgenetic alopecia [9]. Given the multifactorial nature of hair disorders, botanical extracts that influence several of these biological nodes may provide useful experimental leads, provided that their mechanisms are interpreted cautiously and their chemical composition is adequately characterized.

*Eucommia ulmoides* bark extract (EUE), derived from the bark of Du Zhong, is a traditional medicinal plant extract containing lignans, iridoids, flavonoids, phenolic acids, and other phytochemicals with reported antioxidant and anti-inflammatory activities [10,11]. Components of *Eucommia ulmoides*, including geniposidic acid and related phenolic constituents, have been reported to participate in antioxidant or cytoprotective responses [12]. Several studies have also linked Wnt-related signaling to melanocyte and follicle biology [6,7,13]. However, the biological effects of crude botanical extracts should be distinguished from those of purified compounds, because matrix effects, differential permeability, and compound-specific response factors can influence both analytical and biological readouts.

A previous study from our group showed that EUE attenuated oxidative stress and promoted melanogenesis in B16 melanoma cells and mice, accompanied by changes in Wnt/β-catenin-related proteins [14]. Building on that melanogenesis-focused work, the present study evaluates independent datasets related to redox regulation, dermal papilla cell responses, 5α-reductase inhibition, Franz-cell permeation, and H_2_O_2_-induced hair follicle suppression. Accordingly, the present study treats the measured Nrf2, HO-1, VEGF, β-catenin, and Wnt5a changes as exploratory marker observations and does not infer pathway activation from these data.

## 2. Results

### 2.1. Chemical Characterization of EUE by HPLC-DAD

To provide marker-based chemical characterization of the EUE used in the biological assays, HPLC-DAD analysis was performed using geniposidic acid and chlorogenic acid as representative marker compounds. These constituents were detected in EUE by comparison of retention times and online DAD UV absorption spectra with authentic reference standards. The corresponding chromatograms, overlay chromatograms, UV spectra, calibration curves, and quantitative data are provided in the Supplementary Materials. Because EUE is a complex botanical matrix, this analysis supports extract identification and reproducibility but does not by itself identify the bioactive constituent(s) responsible for the observed effects.

The calibration curves showed good linearity for both marker compounds, with correlation coefficients greater than 0.999. Based on the calibration curves, chlorogenic acid and geniposidic acid were quantified at 3.21 ± 0.01 mg/g extract and 1.88 ± 0.15 mg/g extract, respectively. The marker peaks were smaller than several unassigned peaks in the EUE chromatograms, consistent with the multi-component nature of the extract.

Peak height was not used as a direct indicator of absolute content because detector response depends on compound-specific UV absorption characteristics and the selected detection wavelength. Integrated peak areas used for calibration and quantification are therefore provided in the Supplementary Materials to clarify the relationship between chromatographic response and calculated content.

### 2.2. EUE Alters Redox Markers in H_2_O_2_-Challenged B16 Melanoma Cells

**Figure 1:**
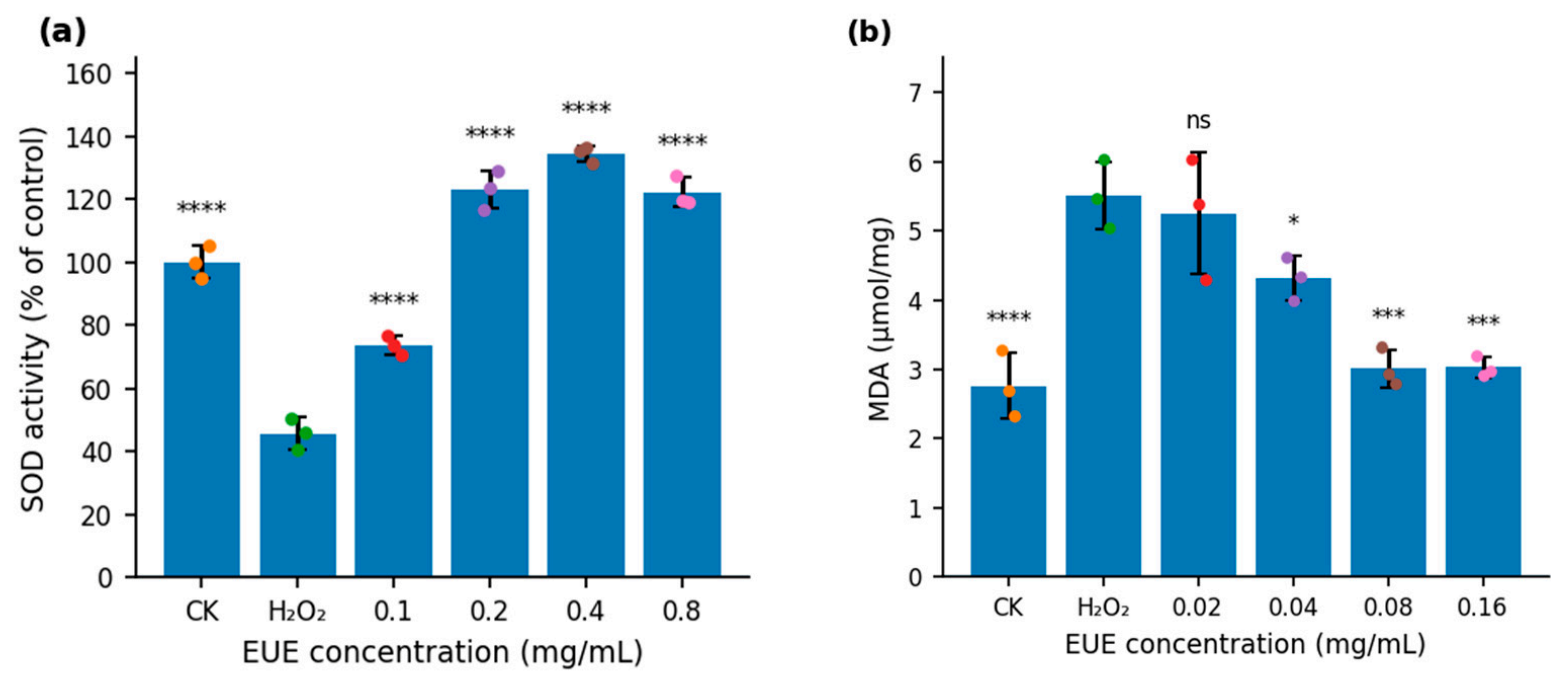
EUE alters antioxidant enzyme activity and lipid peroxidation markers in H_2_O_2_-challenged B16 melanoma cells. (**a**) SOD activity after H_2_O_2_ exposure with or without EUE (0.1–0.8 mg/mL). (**b**) MDA levels after H_2_O_2_ exposure with or without EUE (0.02–0.16 mg/mL). Values are mean ± SD from three separately treated replicate wells within one experiment. Exploratory one-way ANOVA followed by Dunnett’s test was performed against the H_2_O_2_ model group. ns, not significant; * *p* < 0.05; *** *p* < 0.001; **** *p* < 0.0001.

Exposure of B16 melanoma cells to H_2_O_2_ reduced mean SOD activity from 100.0% to 45.7% of the control value, corresponding to a 54.3% reduction (Figure 1a). Mean MDA increased from 2.77 to 5.51 μmol/mg, corresponding to a 1.99-fold increase (Figure 1b). EUE increased SOD activity, with the highest mean observed at 0.4 mg/mL, and reduced MDA, with the lowest mean observed at 0.08 mg/mL. The two assays used independently optimized EUE concentration ranges because the endpoints differed in sensitivity. The values were obtained from three separately treated replicate wells within one experiment; the associated inferential analyses are therefore exploratory.

### 2.3. Exploratory Changes in Nrf2 and HO-1 Markers

**Figure 2:**
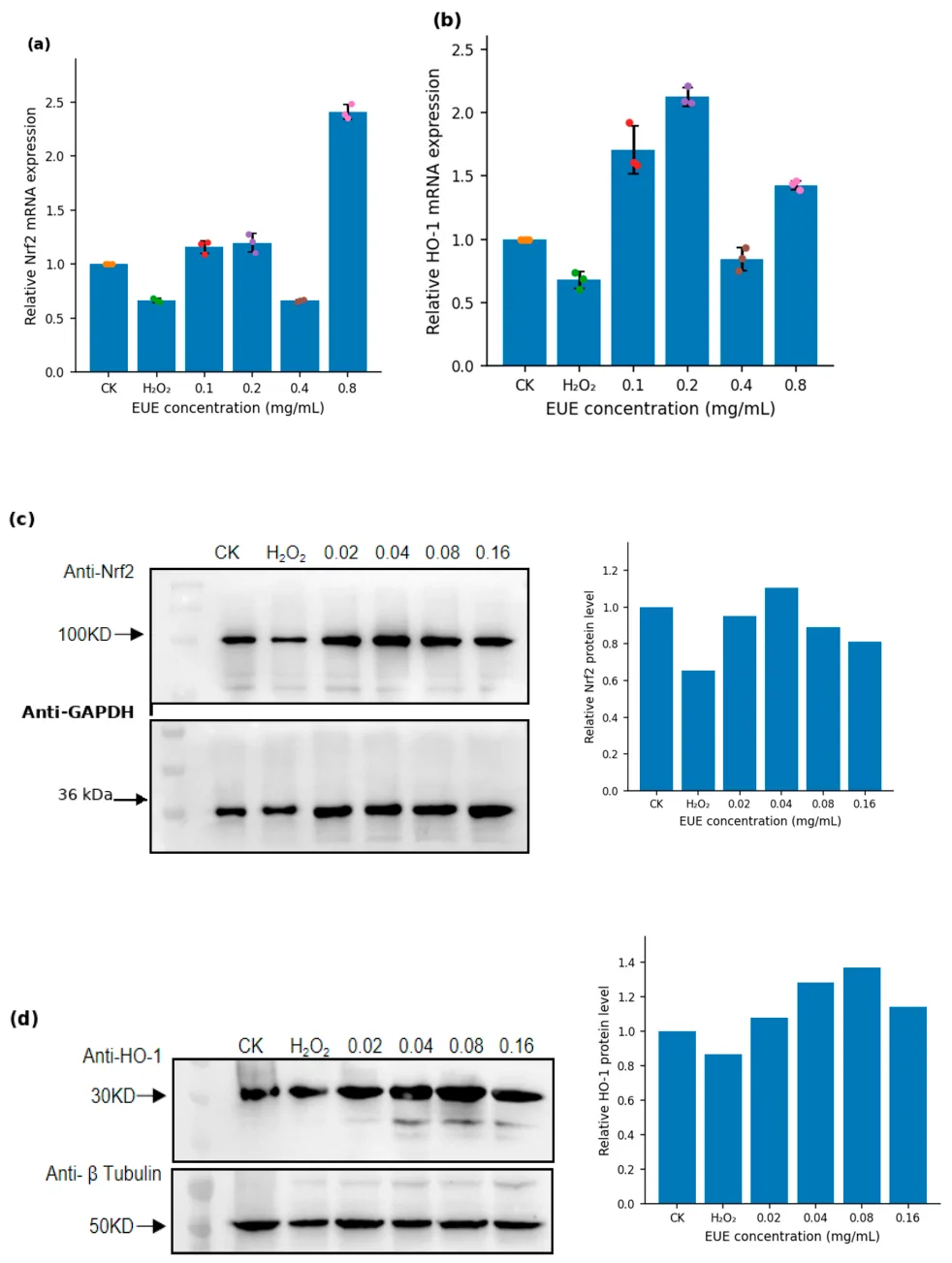
Exploratory assessment of Nrf2 and HO-1 markers in B16 melanoma cells. (**a**,**b**) Relative Nrf2 (**a**) and HO-1 (**b**) mRNA measurements after H_2_O_2_ challenge and EUE treatment (0.1–0.8 mg/mL). Bars show mean ± SD of technical triplicate PCR wells from one cDNA preparation per condition; no inferential statistics were performed. (**c**,**d**) Representative single Western blots for Nrf2 with GAPDH ((**c**); expected GAPDH mass approximately 36 kDa) and HO-1 with β-tubulin (**d**), followed by descriptive normalized densitometry from the displayed blots. No error bars or inferential statistics are shown for the Western blot data. The RT-qPCR and Western blot assays used independently selected EUE concentration ranges and are not interpreted as matched dose–response experiments.

Nrf2 and HO-1 were examined by RT-qPCR and Western blotting (Figure 2). In the RT-qPCR experiment, Figure 2a shows Nrf2 and Figure 2b shows HO-1. The technical-triplicate means were lower after H_2_O_2_ exposure; the highest Nrf2 mean occurred at 0.8 mg/mL EUE, whereas the highest HO-1 mean occurred at 0.2 mg/mL. These measurements were obtained from one cDNA preparation per condition and therefore describe technical variation only. Figure 2c,d show the single Nrf2/GAPDH and HO-1/β-tubulin blots, respectively, together with descriptive normalized densitometry. No inferential statistics were applied to the RT-qPCR or Western blot data. The mRNA and protein assays used independently selected concentration ranges and are presented as exploratory observations of Nrf2 and HO-1 markers, not as a matched transcript-protein dose–response analysis or evidence of pathway activation.

### 2.4. HDPC Viability and Exploratory Folliculogenic Transcript Measurements

**Figure 3:**
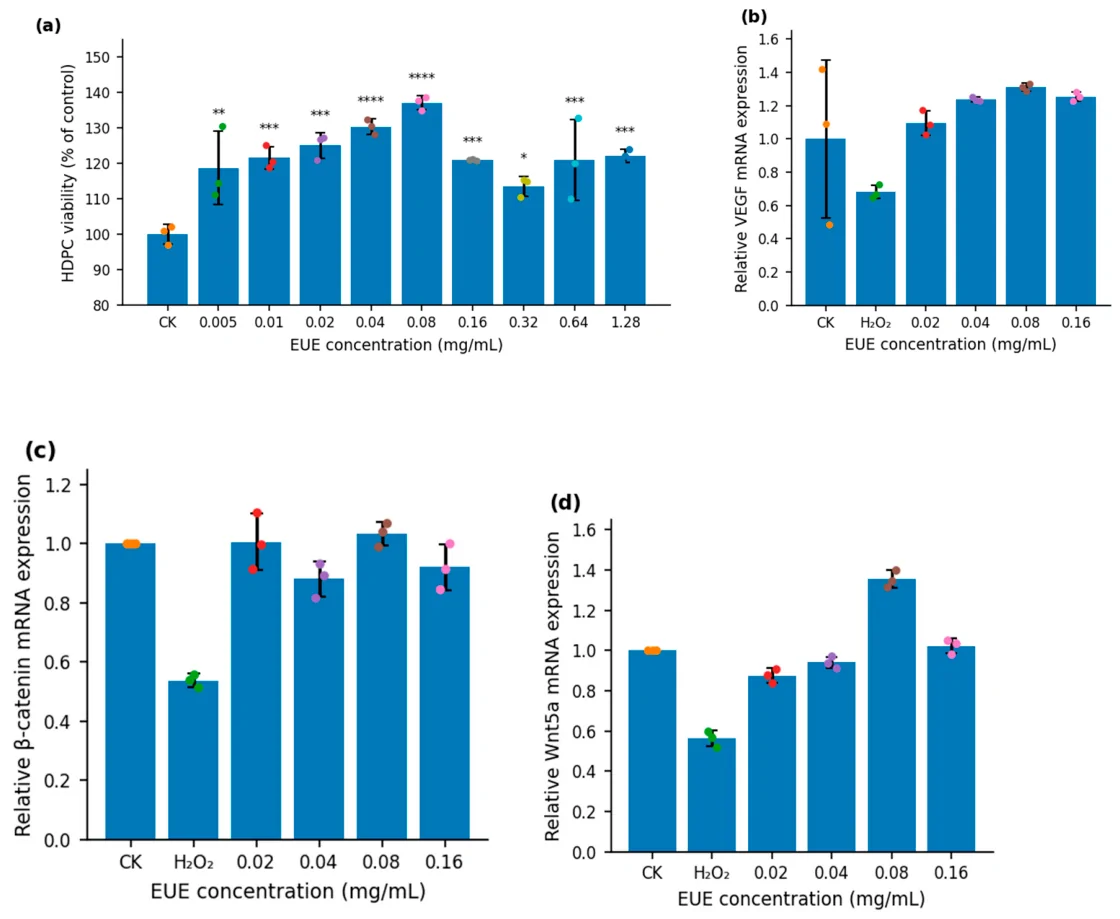
HDPC viability screening and exploratory folliculogenic transcript measurements. (**a**) HDPC viability after 24 h EUE treatment (0.005–1.28 mg/mL). Values are mean ± SD of three replicate wells within one experiment. Exploratory one-way ANOVA followed by Dunnett’s test was performed against the untreated control; * *p* < 0.05; ** *p* < 0.01; *** *p* < 0.001; **** *p* < 0.0001. (**b**–**d**) Relative VEGF (**b**), β-catenin (**c**), and Wnt5a (**d**) mRNA measurements in H_2_O_2_-challenged HDPCs after EUE treatment (0.02–0.16 mg/mL). Bars show mean ± SD of technical triplicate PCR wells from one cDNA preparation per condition. Panels (**b**–**d**) are descriptive; no inferential statistics were performed.

The HDPC viability screen covered 0.005–1.28 mg/mL EUE (Figure 3a). No reduction in mean viability was observed at concentrations up to 0.32 mg/mL, and the highest mean was observed at 0.08 mg/mL. The narrower range of 0.02–0.16 mg/mL was selected for subsequent assays to remain within a conservative non-cytotoxic range and to reduce potential matrix effects from the crude extract. Figure 3a represents three separately treated wells from a single CCK-8 experiment; its inferential analysis is exploratory.

Figure 3b–d show technical-triplicate RT-qPCR measurements from one cDNA preparation per treatment condition in H_2_O_2_-challenged HDPCs. The technical-replicate means for VEGF (Figure 3b), β-catenin (Figure 3c), and Wnt5a (Figure 3d) were higher at selected EUE concentrations than in the H_2_O_2_ model condition, with the highest mean for each endpoint observed at 0.08 mg/mL. Because these were technical rather than independent biological replicates, no inferential statistics were applied. The observations are presented descriptively and require confirmation in independently repeated experiments and at the protein level.

### 2.5. EUE Inhibits 5α-Reductase Activity and Partially Protects HDPCs from DHT-Induced Injury

**Figure 4:**
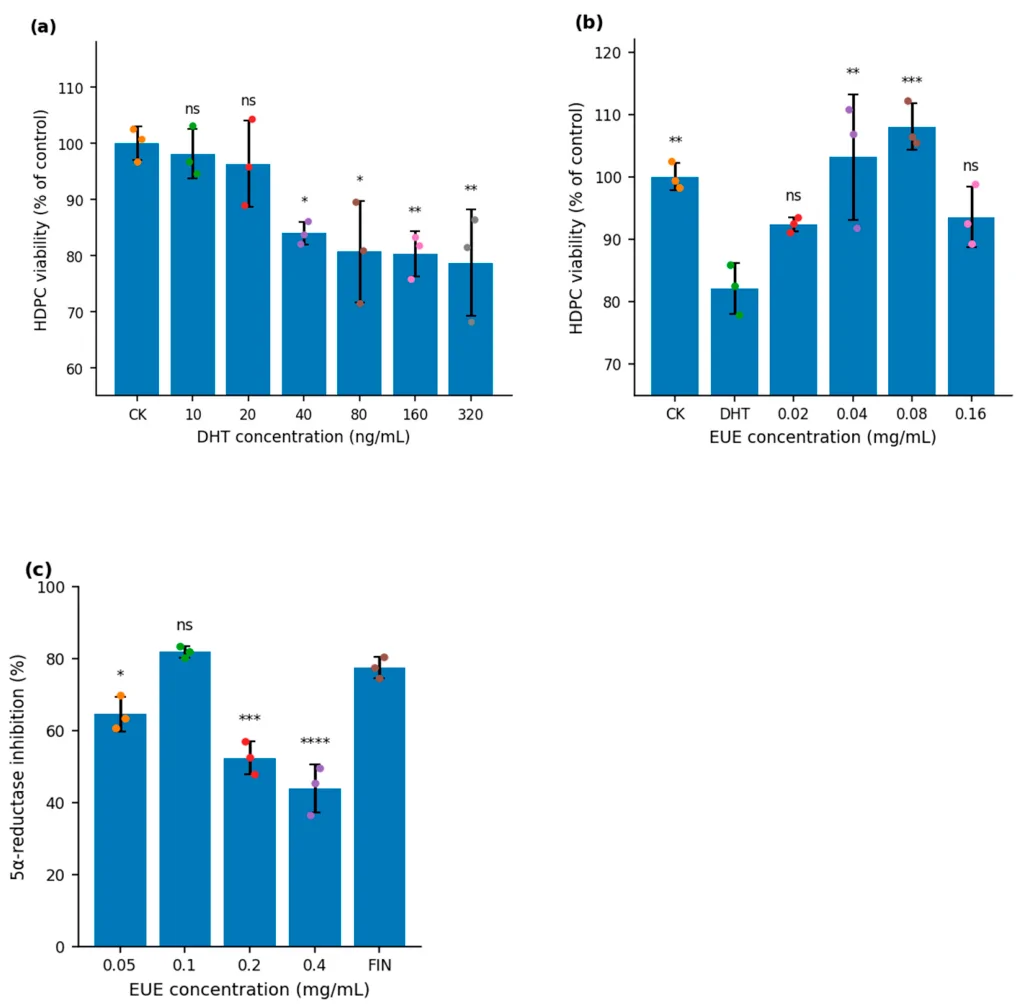
DHT-associated HDPC viability and cell-free 5α-reductase inhibition. (**a**) HDPC viability after 48 h DHT exposure (10–320 ng/mL); exploratory Dunnett comparisons were made against the untreated control. (**b**) HDPC viability after 24 h EUE pretreatment followed by 40 ng/mL DHT; exploratory Dunnett comparisons were made against the DHT-only model group. Panels (**a**,**b**) show mean ± SD of three replicate wells within one experiment. (**c**) 5α-reductase inhibition in three independently prepared reaction tubes. Finasteride (FIN, 0.05 μg/mL) was the declared comparator for Dunnett’s test. Values are mean ± SD. ns, not significant; * *p* < 0.05; ** *p* < 0.01; *** *p* < 0.001; **** *p* < 0.0001.

EUE was evaluated in a cell-free 5α-reductase type II assay using rat prostatic homogenate (Figure 4c). Mean inhibition was 64.7%, 82.0%, 52.5%, and 43.9% at 0.05, 0.1, 0.2, and 0.4 mg/mL EUE, respectively; finasteride (0.05 μg/mL) produced 77.6% inhibition. Dunnett comparisons against finasteride yielded *p* = 0.0168, *p* = 0.5953, *p* = 0.0001, and *p* < 0.0001 for the four EUE concentrations, respectively. These comparisons describe differences under the assay conditions and do not imply potency equivalence, because EUE is a crude extract tested at much higher mass concentrations. The non-monotonic response also precludes reliable IC50 estimation.

In the DHT concentration-screening experiment (Figure 4a), 40–320 ng/mL DHT reduced mean HDPC viability relative to the untreated control, and 40 ng/mL was selected as the challenge concentration for the separate protection experiment. In Figure 4b, the contemporaneous DHT-only group showed a mean viability of 82.1% of control. EUE at 0.04 and 0.08 mg/mL increased mean viability to 103.1% (*p* = 0.0012) and 108.0% (*p* = 0.0002), respectively, relative to the DHT model group, with the strongest mean response at 0.08 mg/mL; 0.02 and 0.16 mg/mL were not significant. Figure 4a,b each represent three replicate wells within one experiment, and the associated analyses are exploratory.

### 2.6. Qualitative Franz-Cell Permeation Assessment of EUE

Franz-cell permeation was assessed as an exploratory topical-feasibility experiment using three diffusion cells containing three dorsal-skin specimens obtained from the same rat. Cumulative UV-absorbing material detected at 280 nm over 24 h was 76.44 ± 3.54%, 48.01 ± 1.99%, and 36.49 ± 2.60% at applied EUE concentrations of 0.5, 1.0, and 2.0 mg/mL, respectively. Because the assay monitored total A280 rather than HPLC-defined markers and the skin specimens were derived from one animal, the values are descriptive and should not be interpreted as compound-specific permeability or as independent-animal replication.

Because EUE is a complex extract, absorbance at 280 nm reflects multiple aromatic and phenolic constituents rather than a single defined analyte. The Franz-cell assay is therefore interpreted only as a qualitative screening experiment. Direct dermal deposition was not measured, and excised rat skin can differ substantially from human scalp skin in permeability. Future studies should track geniposidic acid, chlorogenic acid, or other defined markers by HPLC or LC-MS and should directly quantify skin retention.

### 2.7. EUE Attenuates H_2_O_2_-Induced Hair Follicle Suppression In Vivo

**Figure 5:**
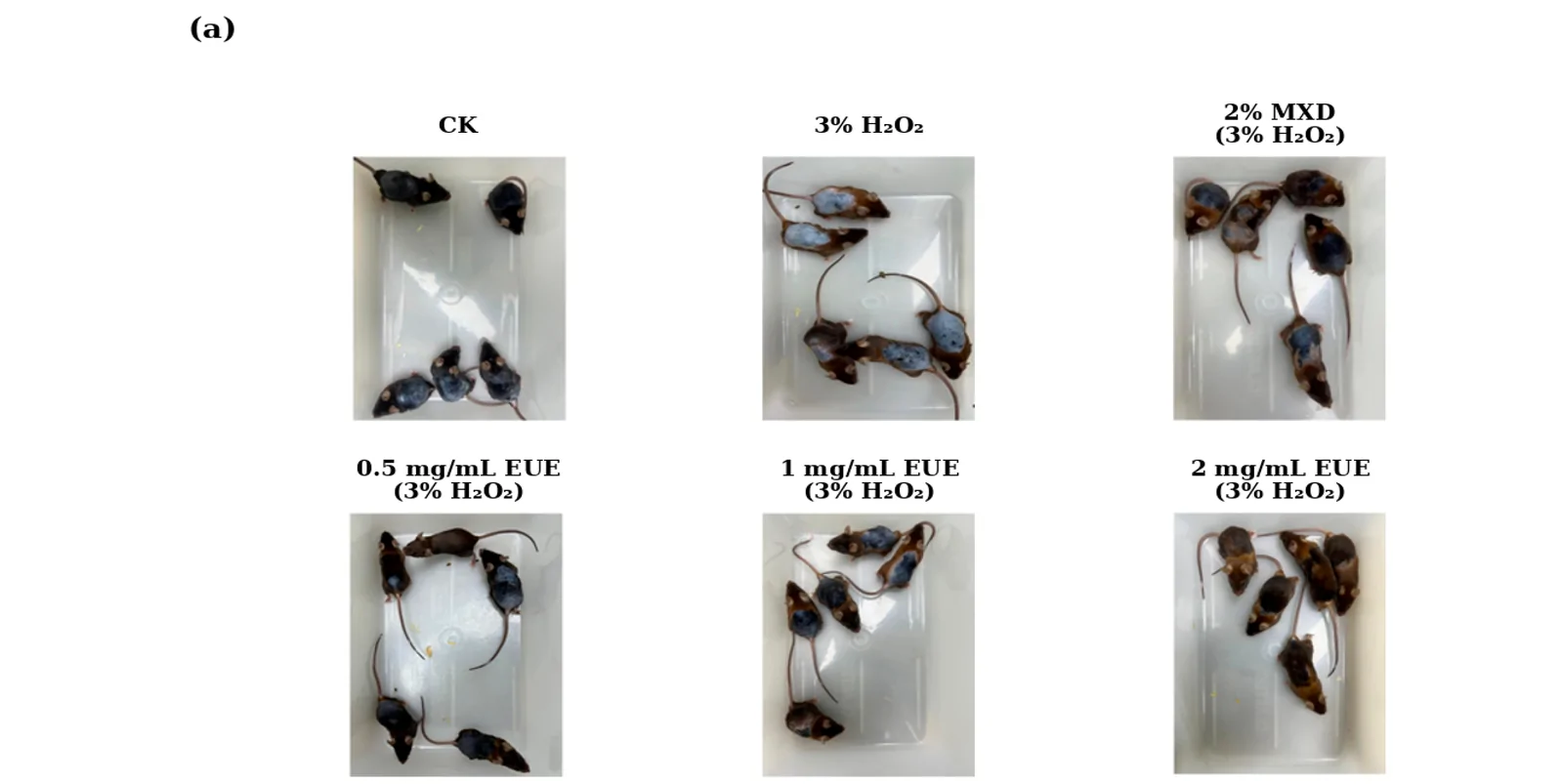

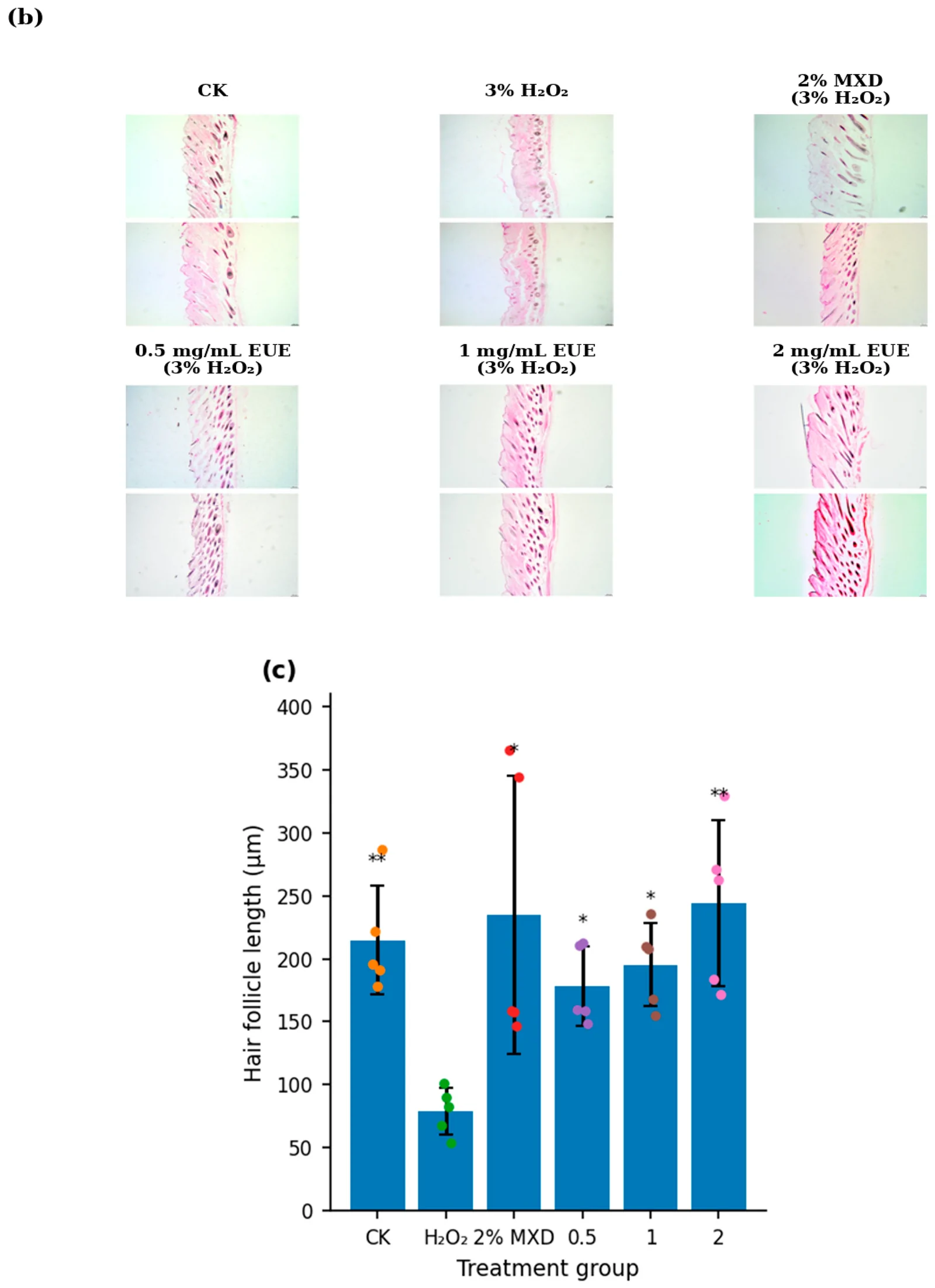
Topical EUE attenuates H_2_O_2_-induced hair follicle suppression in C57BL/6 mice. (**a**) Representative dorsal photographs at day 14; all displayed intervention groups share the H_2_O_2_-challenged background. (**b**) Representative H&E-stained dorsal-skin sections at day 31. Scale bar = 100 μm. (**c**) Animal-level hair follicle length at day 31. For each mouse, two non-consecutive sections were examined and three measurable longitudinal follicles were selected in total across the two sections. Two observers blinded to group allocation measured the same follicles independently; observer values were averaged, followed by calculation of one mean per mouse. Data are mean ± SD, *n* = 5 mice per group. Kruskal–Wallis testing followed by Dunn’s comparisons with Holm adjustment was performed against the H_2_O_2_-only group. * *p* < 0.05; ** *p* < 0.01.

Topical EUE was evaluated in a C57BL/6 oxidative-stress model adapted from our previous in-house topical H_2_O_2_ protocols [14,15]. Distilled water served as the unperturbed baseline, whereas the minoxidil and EUE groups received 3% H_2_O_2_ in the morning and the assigned intervention in the evening. The design therefore evaluates attenuation of H_2_O_2_-induced follicular suppression rather than hair-growth promotion above an unchallenged baseline. Representative day-14 photographs showed visibly greater regrowth in several intervention groups than in the H_2_O_2_-only group (Figure 5a). At day 31, animal-level follicle-length data were non-normally distributed in one group; Kruskal–Wallis testing followed by Dunn’s comparisons with Holm adjustment showed longer follicles in the vehicle control, 2% minoxidil, and all three EUE groups than in the H_2_O_2_-only group (Figure 5c). The response was variable and not monotonic across EUE concentrations.

Representative H&E sections showed deeper and more developed follicular structures in EUE-treated mice than in the H_2_O_2_-only group (Figure 5b). Because no EUE-only group was included, the findings remain limited to attenuation of oxidative stress-induced follicular suppression and do not establish hair-growth promotion under unchallenged conditions.

## 3. Discussion

This study evaluates EUE as a chemically characterized botanical extract with preliminary activities relevant to hair follicle biology. HPLC-DAD identified geniposidic acid and chlorogenic acid as representative markers, while the biological experiments examined redox endpoints, descriptive measurements of Nrf2, HO-1, VEGF, β-catenin, and Wnt5a markers, HDPC viability and transcripts, cell-free 5α-reductase inhibition, qualitative Franz-cell permeation, and an animal model of H_2_O_2_-induced follicular suppression. The strength of inference differs among these datasets: the animal analysis used mice as the biological unit, whereas several cell-based measurements were generated from within-experiment replicate wells, technical PCR replicates, or a single Western blot and must therefore be interpreted as exploratory.

### 3.1. Redox and Folliculogenic Observations

H_2_O_2_ reduced SOD activity by 54.3% and increased MDA approximately 1.99-fold in the present replicate-well experiment, while EUE shifted both endpoints toward the control range. This direction is consistent with reported antioxidant activity of E. ulmoides extracts, polysaccharides, and flavonoid-rich fractions [11,12,16,17], as well as our previous observation that EUE reduced ROS accumulation in H_2_O_2_-challenged B16 cells [14]. Chlorogenic acid and geniposidic acid provide useful markers for extract characterization, and both phenolic acids and iridoids have been associated with antioxidant or cytoprotective effects; nevertheless, the marker peaks represented only part of a complex chromatographic profile. The current data therefore cannot attribute the redox effects to either marker or exclude contributions from lignans, flavonoids, polysaccharides, or unassigned constituents. The Nrf2/HO-1 measurements further remain descriptive because RT-qPCR used technical replicates and Western blotting was performed once; the established role of Nrf2 in antioxidant defense in skin cells provides relevant mechanistic context [18]. Independent biological replication, nuclear Nrf2 assessment, and downstream targets such as NQO1 and GCLC are required.

The HDPC viability screen showed its largest mean response at 0.08 mg/mL EUE, while the technical-replicate RT-qPCR means for VEGF, β-catenin, and Wnt5a also peaked at 0.08 mg/mL. VEGF is relevant to perifollicular angiogenesis [8], and β-catenin-associated signaling contributes to follicle morphogenesis and cycling [6,7]. Wnt5a, however, is often associated with non-canonical Wnt signaling, so its increase cannot be taken as proof of canonical β-catenin activation. Our previous B16 study detected Wnt-related protein changes [14], but those results do not substitute for HDPC-specific validation. The present transcript observations are based on one cDNA preparation per condition and should be regarded as hypothesis-generating until reproduced with independent biological samples and confirmed by VEGF protein, active or nuclear β-catenin, AXIN2/LEF1, or pathway-reporter assays.

### 3.2. Antiandrogenic Action and Topical Feasibility

EUE showed measurable but non-monotonic inhibition of 5α-reductase type II in a cell-free rat prostatic homogenate assay. The 0.1 mg/mL mean was closest to the finasteride control, whereas higher extract concentrations produced lower apparent inhibition. Such a pattern may reflect matrix interference, solubility limitations, or counteracting constituents in the crude extract and does not support an IC50 estimate or a potency comparison with finasteride. In the separate DHT-HDPC experiment, the highest mean viability occurred at 0.08 mg/mL EUE, but the result was derived from replicate wells within one experiment and remains preliminary.

The Franz-cell experiment should be considered an exploratory topical-feasibility screen. Because total absorbance at 280 nm aggregates multiple UV-absorbing constituents, it cannot define the permeation of geniposidic acid, chlorogenic acid, or any other individual marker. Moreover, direct skin deposition was not measured, and rat skin can overestimate permeability relative to human skin. The current results therefore justify further formulation and marker-specific permeation studies but do not establish follicular bioavailability.

### 3.3. Limitations and Future Directions

Several limitations should be considered. First, marker identification relied on retention time and DAD spectral similarity rather than standard addition or MS confirmation, and the two quantified markers do not identify the active constituent(s) in a complex extract. Second, several in vitro datasets were generated from replicate wells within one experiment. The RT-qPCR measurements were technical triplicates from one cDNA preparation per condition, and the Western blot was performed once; unsupported inferential statistics were therefore removed from these datasets. Third, no dedicated HDPC H_2_O_2_ dose-viability curve was generated, although the selected exposure falls within concentrations previously evaluated in human dermal papilla cells [19]. Fourth, the mouse study lacked an EUE-only group, so conclusions are limited to attenuation of H_2_O_2_-induced suppression. Fifth, the Franz assay used total A280 and three skin specimens from one rat, precluding analyte-specific or between-animal inference. Finally, the study lacked an antioxidant positive control, in-tissue validation of the measured markers, post-receipt STR authentication, and formal mycoplasma testing. These limitations substantially constrain mechanistic and generalizable conclusions.

Future studies should focus on: (1) activity-guided fractionation and isolation of lead compounds followed by target validation; (2) standard-addition or LC-MS confirmation of chemical markers; (3) marker-specific skin permeation and direct dermal-deposition assays; (4) protein-level validation of VEGF, active/nuclear β-catenin, and canonical Wnt targets in HDPCs; (5) in-tissue validation of Nrf2, HO-1, and β-catenin in mouse skin; and (6) early-stage safety and proof-of-concept testing in relevant human models.

## 4. Materials and Methods

### 4.1. Chemicals and Reagents

B16 mouse melanoma cells were purchased from Shanghai Zhong Qiao Xin Zhou Biotechnology Co., Ltd. (Shanghai, China; catalogue ZQ0186; original depositor: JCRB Cell Bank, JCRB0202). Human dermal papilla cells (HDPCs) were purchased from the same supplier (Shanghai, China; catalogue ZQY002; supplier-reported original source: ScienCell Research Laboratories, Cat. No. 2400). Both cell types were used at passages 3–6. Lot numbers were not recorded in the retained laboratory documentation. Dulbecco’s modified Eagle’s medium (DMEM), fetal bovine serum (FBS), penicillin, and streptomycin were purchased from Gibco, Thermo Fisher Scientific (Waltham, MA, USA). Cells were cultured in DMEM supplemented with 10% FBS, 100 U/mL penicillin, and 100 μg/mL streptomycin at 37 °C in a humidified atmosphere containing 5% CO_2_. Anti-GAPDH antibody was purchased from Affinity Biosciences (Changzhou, China; Cat. No. AF7021; expected molecular mass approximately 36 kDa). EUE was supplied by Shanghai Zhina Biotechnology Co., Ltd. (Shanghai, China) as a commercially prepared dried E. ulmoides bark extract powder. Detailed proprietary extraction parameters were unavailable; the extract was therefore characterized by high-performance liquid chromatography coupled with diode array detection (HPLC-DAD). No post-receipt STR authentication or formal mycoplasma testing was performed.

### 4.2. HPLC-DAD Characterization of EUE

EUE was chemically characterized by HPLC-DAD using geniposidic acid and chlorogenic acid as representative marker compounds. HPLC analysis was performed using a system equipped with a diode-array detector on a reversed-phase C18 column (4.6 × 250 mm, 5 μm) maintained at 30 °C. The mobile phase consisted of 0.1% phosphoric acid in water (A) and acetonitrile (B), delivered at a flow rate of 1.0 mL/min with gradient elution. The injection volume was 10 μL. Detection was performed at 240 nm for geniposidic acid and 325 nm for chlorogenic acid, and full online DAD spectral acquisition was performed from 190 to 400 nm.

Reference standards of geniposidic acid and chlorogenic acid were prepared in methanol and used to establish calibration curves. EUE powder was extracted with 50% methanol by ultrasonication for 30 min, cooled to room temperature, adjusted to volume, and filtered through a 0.22 μm membrane before injection. Marker identification was supported by comparison of retention times and online DAD UV spectra with authentic standards. Marker contents were calculated from the corresponding calibration curves and expressed as mg/g extract. Detailed chromatograms, UV spectra, integrated peak areas, calibration curves, and quantitative tables are provided in the Supplementary Materials.

### 4.3. Cell Proliferation Assay (CCK-8)

HDPCs were cultured to 80% confluence, trypsinized, and seeded at 1 × 10^4^ cells/well in 96-well plates. After 24 h, cells were treated with EUE (0.005–1.28 mg/mL) for 24 h. CCK-8 reagent (10%) was then added for 4 h, and absorbance was measured at 450 nm. Three separately seeded and treated wells were analyzed for each condition within one experiment. Concentrations up to 0.32 mg/mL did not reduce mean viability; 0.02–0.16 mg/mL was selected for subsequent assays as a conservative range intended to reduce high-dose matrix effects.

### 4.4. Antioxidant Assays in H_2_O_2_-Challenged B16 Melanoma Cells

B16 melanoma cells were assigned to untreated control, H_2_O_2_ model, and EUE-treatment conditions. EUE concentrations of 0.1–0.8 mg/mL were used for SOD analysis and 0.02–0.16 mg/mL for MDA analysis because the biochemical endpoints had been optimized separately. After EUE pre-incubation, H_2_O_2_ was added to induce oxidative stress. SOD activity was measured using a WST-1-based assay at 450 nm [20], and MDA was measured using the thiobarbituric-acid method at 532 nm [21]. Three separately cultured and treated wells were analyzed per condition within one experiment. The selected B16 concentration range was supported by prior cytotoxicity data showing acceptable viability up to 0.8 mg/mL EUE [14]. No antioxidant positive control was included.

### 4.5. DHT-Induced HDPC Injury Model

HDPCs were incubated with DHT (10–320 ng/mL) for 48 h in the concentration-screening experiment. For the separate protection experiment, cells were pretreated with EUE (0.02, 0.04, 0.08, or 0.16 mg/mL) for 24 h and then co-incubated with 40 ng/mL DHT for 48 h. Viability was assessed by CCK-8 using three separately treated wells per condition within each experiment.

### 4.6. Inhibition of 5α-Reductase Activity

Prostate tissue from 8-week-old male Sprague-Dawley rats was homogenized in sucrose buffer and centrifuged to obtain enzyme fractions. Three independently prepared reaction tubes were analyzed per condition. Reaction mixtures containing testosterone, NADPH, and EUE were incubated at 37 °C for 2 h, terminated with dichloromethane, evaporated under nitrogen, and analyzed by LC-MS/MS to quantify testosterone and DHT [22,23]. Finasteride (0.05 μg/mL) served as the positive control and statistical comparator. Inhibition (%) was calculated as (1 − DHTsample/DHTcontrol) × 100% [9].

### 4.7. RT–qPCR Analysis

RNA was extracted using TRIzol reagent and reverse-transcribed into cDNA. RT-qPCR was performed using SYBR Green Master Mix, with GAPDH as the internal control and relative expression calculated by the 2^−ΔΔCt^ method [24]. Nrf2 and HO-1 were measured in B16 melanoma cells, whereas VEGF, β-catenin, and Wnt5a were measured in HDPCs. For each condition, one cDNA preparation was analyzed in three PCR wells; these were technical triplicates and not independent biological replicates. For the HDPC oxidative-stress experiment, cells were exposed to 0.05 mg/mL H_2_O_2_ (approximately 1.47 mM) for 0.5 h, washed twice with PBS, and then treated with EUE (0.02–0.16 mg/mL) in fresh medium for 48 h. The H_2_O_2_ condition was adapted from our B16 protocol [14] and falls within the 250–2000 μM range evaluated in cultured human dermal papilla cells; 1 mM for 30 min has also been used for an acute ROS assay [19]. A dedicated HDPC H_2_O_2_ dose-viability curve was not generated in the present study. Primer details are provided in Supplementary Table S1.

### 4.8. Western Blot Analysis

B16 melanoma cells were lysed on ice in protease-inhibitor-containing buffer. Equal protein amounts were separated by SDS-PAGE and transferred to PVDF membranes. After blocking with 5% milk, membranes were incubated overnight at 4 °C with antibodies against Nrf2 or HO-1 and the corresponding loading control. Nrf2/GAPDH and HO-1/β-tubulin were analyzed on separate membranes. HRP-conjugated secondary antibodies were applied for 2 h, signals were visualized using ECL, and band intensity was measured with ImageJ 1.54f [25]. One blot was generated for each target/loading-control set. Repeated ImageJ readings of the same bands were not treated as independent replicates; the revised figure therefore presents only descriptive normalized densitometry without error bars or inferential statistics.

### 4.9. Franz Diffusion Assay

The percutaneous permeation of EUE was evaluated using a Franz diffusion-cell system with excised rat dorsal skin [26,27]. Three diffusion cells were fitted with three separate skin specimens obtained from the same rat. The donor chamber contained 2 mL EUE solution (0.5, 1.0, or 2.0 mg/mL), and the receptor chamber contained 15 mL PBS (pH 7.4) maintained at 37 °C with continuous stirring. Receptor samples were collected at 2, 4, 8, 12, and 24 h and replaced with equal volumes of fresh PBS. Absorbance at 280 nm was used as an index of total UV-absorbing constituents rather than a single analyte. A calibration curve of EUE in PBS was used for concentration calculation, and cumulative permeation was expressed relative to the applied dose. Because all three skin specimens came from one rat, the three values represent diffusion units rather than independent animals. The cumulative permeation of EUE through rat skin after 24 h was summarized in Table 1.

### 4.10. In Vivo H_2_O_2_-Induced Follicular Suppression Model

Seven-week-old male C57BL/6 mice were acclimated for one week and depilated over a 2 × 2 cm dorsal area. The topical oxidative-stress design was adapted from our previous 3% H_2_O_2_ mouse protocols [14,15]. Mice were randomly assigned to six groups (*n* = 5/group; the previously reported *n* = 6 was a transcription error). No formal a priori power calculation was performed; the group size of five mice was selected based on our previous similar C57BL/6 topical H_2_O_2_ studies [14,15] and practical experience with this model. The six groups were: (1) vehicle control, distilled water in the morning and evening; (2) H_2_O_2_ model, 200 μL 3% H_2_O_2_ in the morning and 200 μL distilled water in the evening; (3) positive control, 200 μL 3% H_2_O_2_ in the morning and 200 μL 2% minoxidil in the evening; and (4–6) EUE groups, 200 μL 3% H_2_O_2_ in the morning and 200 μL EUE at 0.5, 1.0, or 2.0 mg/mL in the evening. Treatment continued for 30 days. The EUE concentrations matched the range examined in the Franz-cell screen and were below or comparable to topical concentrations used in our prior in-house studies [14,15]. No ulceration, overt skin breakdown, or systemic adverse signs were observed. Mice were euthanized on day 31, and dorsal skin was fixed, paraffin-embedded, and H&E-stained. For each mouse, two non-consecutive sections were examined; three well-oriented, longitudinally sectioned follicles with clearly visible bulbs were selected in total across the two sections. Group allocation and treatment administration were not blinded. For histological outcome assessment, two observers blinded to group allocation independently measured the same follicles from the epidermal surface to the deepest visible bulb region. The two observer measurements were averaged for each follicle, and the three follicle values were then averaged to obtain one animal-level value for statistical analysis. The analyst worked with coded group labels and remained unaware of group identities during data processing and statistical analysis; group identities were revealed after the animal-level analysis was completed.

### 4.11. Statistical Analysis

Data are reported as mean ± SD. Statistical analyses reported in the revised manuscript were performed using Python 3.11 with the SciPy 1.13.0 package. Source records were re-audited to distinguish biological replicates (individual mice), replicate wells within a single experiment, independently prepared reaction tubes, independent Franz diffusion units, technical PCR replicates, and repeated measurements of the same Western blot. Shapiro–Wilk testing was used as a screening assessment of distributional assumptions. For replicate-well and 5α-reductase datasets in which no significant departure from normality was detected, one-way ANOVA followed by Dunnett’s test was used against the comparator declared in each figure legend; because several datasets arose from one experiment, these analyses are exploratory. RT-qPCR technical triplicates and single-blot densitometry were reported descriptively without inferential statistics. Figure 5 animal-level data showed non-normality in one group and were analyzed by Kruskal–Wallis testing followed by Dunn’s comparisons against the H_2_O_2_ group with Holm adjustment. Franz-cell results were reported descriptively. Significance thresholds were ns, not significant; * *p* < 0.05; ** *p* < 0.01; *** *p* < 0.001; and **** *p* < 0.0001.

## 5. Conclusions

HPLC-DAD provided marker-based characterization of EUE and identified geniposidic acid and chlorogenic acid as representative constituents. Replicate-well assays showed preliminary effects on B16 redox endpoints and HDPC viability, while technical-triplicate RT-qPCR and single-blot data provided descriptive, hypothesis-generating observations of the measured Nrf2, HO-1, VEGF, β-catenin, and Wnt5a markers. EUE also showed non-monotonic 5α-reductase inhibition in a cell-free assay and attenuated H_2_O_2_-induced follicular suppression in an animal-level analysis. These findings support further investigation of EUE, but independent biological replication, active-compound identification, marker-specific permeation, protein-level validation, and appropriately powered mechanistic studies are required before firm efficacy or mechanistic conclusions can be drawn.

## Figures and Tables

**Table 1:** Exploratory 24 h Franz-cell permeation assessment of EUE using three independent diffusion cells containing three skin specimens from the same rat (mean ± SD; three diffusion units).

EUE Concentration (mg/mL)	Cumulative Permeation (% ± SD)
0.5	76.44 ± 3.54
1.0	48.01 ± 1.99
2.0	36.49 ± 2.60

## Data Availability

The data presented in this study are available on request from the corresponding author.

## Supplementary Materials

The following supporting information can be downloaded at: https://www.mdpi.com/article/10.3390/molecules31162863/s1, Table S1: The primer sequences, annealing temperatures, and amplification efficiencies; Figure S1: HPLC-DAD chromatograms of reference standards; Figure S2: HPLC-DAD chromatograms of the mixed reference standards containing geniposidic acid and chlorogenic acid; Figure S3: HPLC-DAD chromatograms of EUE showing the geniposidic acid peak at 240 nm in replicate analyses; Figure S4: HPLC-DAD chromatograms of EUE showing the chlorogenic acid peak at 325 nm in replicate analyses; Figure S5: Overlay chromatograms of reference standards and EUE; Figure S6: Online DAD UV absorption spectra of the representative marker compounds; Figure S7: Calibration curves of chlorogenic acid and geniposidic acid used for marker quantification; Table S2: HPLC-DAD identification and quantification of representative marker compounds in EUE; Table S3: Calibration curves and quantitative results for marker compounds in EUE.

## References

[1] Arck P.C., Overall R., Spatz K., Liezman C., Handjiski B., Klapp B.F., Birch-Machin M.A., Peters E.M.J. (2006). Towards a “Free Radical Theory of Graying”: Melanocyte Apoptosis in the Aging Human Hair Follicle Is an Indicator of Oxidative Stress Induced Tissue Damage. FASEB J..

[2] O’Sullivan J.D., Nicu C., Picard M., Chéret J., Bedogni B., Tobin D.J., Paus R. (2021). The Biology of Human Hair Greying. Biol. Rev..

[3] Trüeb R.M. (2009). Oxidative Stress in Ageing of Hair. Int. J. Trichology.

[4] Ngo V., Duennwald M.L. (2022). Nrf2 and Oxidative Stress: A General Overview of Mechanisms and Implications in Human Disease. Antioxidants.

[5] Trüeb R. (2015). The Impact of Oxidative Stress on Hair. Int. J. Cosmet. Sci..

[6] Choi B.Y. (2020). Targeting Wnt/β-Catenin Pathway for Developing Therapies for Hair Loss. Int. J. Mol. Sci..

[7] Veltri A., Lang C., Lien W.-H. (2018). Concise Review: Wnt Signaling Pathways in Skin Development and Epidermal Stem Cells. Stem Cells.

[8] Yano K., Brown L.F., Detmar M. (2001). Control of Hair Growth and Follicle Size by VEGF-Mediated Angiogenesis. J. Clin. Investig..

[9] Robitaille J., Langlois V.S. (2020). Consequences of Steroid-5α-Reductase Deficiency and Inhibition in Vertebrates. Gen. Comp. Endocrinol..

[10] Hussain T., Tan B., Liu G., Oladele O.A., Rahu N., Tossou M., Yin Y. (2016). Health-Promoting Properties of *Eucommia ulmoides*: A Review. Evid.-Based Complement. Altern. Med..

[11] Wang C.-Y., Tang L., He J.-W., Li J., Wang Y.-Z. (2019). Ethnobotany, Phytochemistry and Pharmacological Properties of *Eucommia ulmoides*: A Review. Am. J. Chin. Med..

[12] Xiao D., Yuan D., Tan B., Wang J., Liu Y. (2019). The Role of Nrf2 Signaling Pathway in *Eucommia ulmoides* Flavones Regulating Oxidative Stress in the Intestine of Piglets. Oxid. Med. Cell. Longev..

[13] Clevers H., Nusse R. (2012). Wnt/β-Catenin Signaling and Disease. Cell.

[14] Liu X., Qiu Y., Lv X., Chang L., Ji T., Gu Y., Chen S. (2025). *Eucommia ulmoides* Extract Attenuates Oxidative Stress and Promotes Melanogenesis via Wnt/β-Catenin Signaling in B16 Cells and Mice. Turk. J. Biol..

[15] Liu X., Chang L., Qiu Y., Lv X., Ji T. (2026). The Role of Gynostemma pentaphyllum Extract in Combating H_2_O_2_-Induced Oxidative Stress and Preventing Hair Graying. Dermatol. Res. Pract..

[16] Peng Y., Yang Y., Tian Y., Zhang M., Cheng K., Zhang X., Zhou M., Hui M., Zhang Y. (2024). Extraction, Characterization, and Antioxidant Activity of *Eucommia ulmoides* Polysaccharides. Molecules.

[17] Xu Z., Tang M., Li Y., Liu F., Li X., Dai R. (2010). Antioxidant Properties of Du-Zhong (*Eucommia ulmoides* Oliv.) Extracts and Their Effects on Color Stability and Lipid Oxidation of Raw Pork Patties. J. Agric. Food Chem..

[18] Gęgotek A., Skrzydlewska E. (2015). The Role of Transcription Factor Nrf2 in Skin Cells Metabolism. Arch. Dermatol. Res..

[19] Choi Y.-H., Shin J.Y., Kim J., Kang N.-G., Lee S. (2021). Niacinamide Down-Regulates the Expression of DKK-1 and Protects Cells from Oxidative Stress in Cultured Human Dermal Papilla Cells. Clin. Cosmet. Investig. Dermatol..

[20] Peskin A.V., Winterbourn C.C. (2000). A Microtiter Plate Assay for Superoxide Dismutase Using a Water-Soluble Tetrazolium Salt (WST-1). Clin. Chim. Acta.

[21] Draper H.H., Hadley M. (1990). Malondialdehyde Determination as Index of Lipid Peroxidation. Methods Enzymol..

[22] Gorityala S., Yang S., Montano M.M., Xu Y. (2018). Simultaneous Determination of Dihydrotestosterone and Its Metabolites in Mouse Sera by LC-MS/MS with Chemical Derivatization. J. Chromatogr. B.

[23] Keevil B.G. (2016). LC–MS/MS Analysis of Steroids in the Clinical Laboratory. Clin. Biochem..

[24] Livak K.J., Schmittgen T.D. (2001). Analysis of Relative Gene Expression Data Using Real-Time Quantitative PCR and the 2^−ΔΔCT^ Method. Methods.

[25] Taylor S.C., Posch A. (2014). The Design of a Quantitative Western Blot Experiment. BioMed Res. Int..

[26] Flaten G.E., Palac Z., Engesland A., Filipović-Grčić J., Vanić Ž., Škalko-Basnet N. (2015). In Vitro Skin Models as a Tool in Optimization of Drug Formulation. Eur. J. Pharm. Sci..

[27] Neupane R., Boddu S.H., Renukuntla J., Babu R.J., Tiwari A.K. (2020). Alternatives to Biological Skin in Permeation Studies: Current Trends and Possibilities. Pharmaceutics.

