# Supplementary material for: Eucommia ulmoides Bark Extract Attenuates Oxidative Damage and H2O2-Induced Hair Follicle Suppression in Cellular and Mouse Models
Source: Molecules. 2026 Aug 17;31(16):2863. doi: 10.3390/molecules31162863 (PMC13515758; doi:10.3390/molecules31162863)
Supplement: Supplementary file 1 [file molecules-31-02863-s001.zip › Supplementary Table S1-0811.pdf]

Supplementary Table S1: The primer sequences, annealing temperatures, and amplification efficiencies.

Species	Gene	Forward Primer Sequence (5′–3′)	Reverse Primer Sequence (5′–3′)	Annealing Temp (°C)	Amplification Efficiency (%)
Mouse (B16)	Nrf2	TCTTGGAGTAAG TCGAGAAGTGT	GTTGAAACTGAGCGA AAAAGGC	58.0	98.6
	HO-1	AAGCCGAGAAT GCTGAGTTCA	GCCGTGTAGATATGGT ACAAGGA	58.0	101.2
	GAPDH	TGTTGCCATCAA TGACCCCTT	CTCCACGACGTACTC AGCG	58.0	99.5
Human (HDPC)	VEGF	TCTTCAAGCCAT CCTGTGTG	GCGAGTCTGTGTTTTT GCAG	60.0	97.8
	β-catenin	AACTTGCTCAGG ACAAGGAA	TCCTAAAGGATGATTT ACAGGTC	60.0	102.1
	Wnt5a	TTGAAGCCAATT CTTGGTGGTCGC	TGGTCCTGATACAAG TGGCACAGT	60.0	99.1
	GAPDH	CAATGACCCCTT CATTGACC	GACAAGCTTCCCGTT CTCAG	60.0	100.4
