# Supplementary material for: Eucommia ulmoides Bark Extract Attenuates Oxidative Damage and H2O2-Induced Hair Follicle Suppression in Cellular and Mouse Models
Source: Molecules. 2026 Aug 17;31(16):2863. doi: 10.3390/molecules31162863 (PMC13515758; doi:10.3390/molecules31162863)
Supplement: Supplementary file 1 [file molecules-31-02863-s001.zip › Supplementary_Materials_EUE_HPLC-DAD-0811.pdf]

Supplementary Materials

HPLC-DAD characterization of Eucommia ulmoides bark extract

Chemical characterization of *Eucommia ulmoides* bark extract (EUE). To provide marker-based chemical characterization of the EUE used in the biological assays, HPLC-DAD analysis was performed using geniposidic acid and chlorogenic acid as representative marker compounds. The aim of this analysis was to support the reproducibility and chemical traceability of the extract, rather than to claim complete identification of all bioactive constituents in EUE.

Note: Supplementary Table numbering in this file assumes that Supplementary Table S1 is reserved for RT-qPCR primer information in the full supplementary package.

HPLC-DAD method

EUE and reference standards were analyzed using an HPLC system equipped with a diode-array detector (DAD). Separation was performed on a reversed-phase C18 column (4.6 × 250 mm, 5 μm) at 30 °C. The mobile phase consisted of solvent A (0.1% phosphoric acid in water) and solvent B (acetonitrile), using gradient elution. The flow rate was 1.0 mL/min and the injection volume was 10 μL. DAD signals were monitored at 240 nm for geniposidic acid and at 325 nm for chlorogenic acid, with full spectral acquisition over 190–400 nm.

For sample preparation, EUE powder was dissolved/extracted in 50% methanol by ultrasonication for 30 min, cooled to room temperature, adjusted to volume, and filtered through a 0.22 μm membrane before injection. Marker compounds were identified by comparison with authentic standards based on retention time and online DAD spectral characteristics.

Summary of marker identification and quantification

Table S2. HPLC-DAD identification and quantification of representative marker compounds in EUE.

Compound	Detection wavelength	Retention time in standards	Retention time in EUE	Content in EUE
Geniposidic acid	240 nm	9.172–9.473 min	9.151–9.354 min	1.88 ± 0.15 mg/g extract
Chlorogenic acid	325 nm	11.717–11.804 min	11.746–12.075 min	3.21 ± 0.01 mg/g extract

Note. The values represent marker-based characterization of the extract. The contents were calculated from the corresponding calibration curves and are reported as mean ± SD.

Chromatographic characterization

Figure S1. HPLC-DAD chromatograms of reference standards. (A) Geniposidic acid single standard (100 μg/mL, 240 nm). (B) Chlorogenic acid single standard (100 μg/mL, 325 nm).

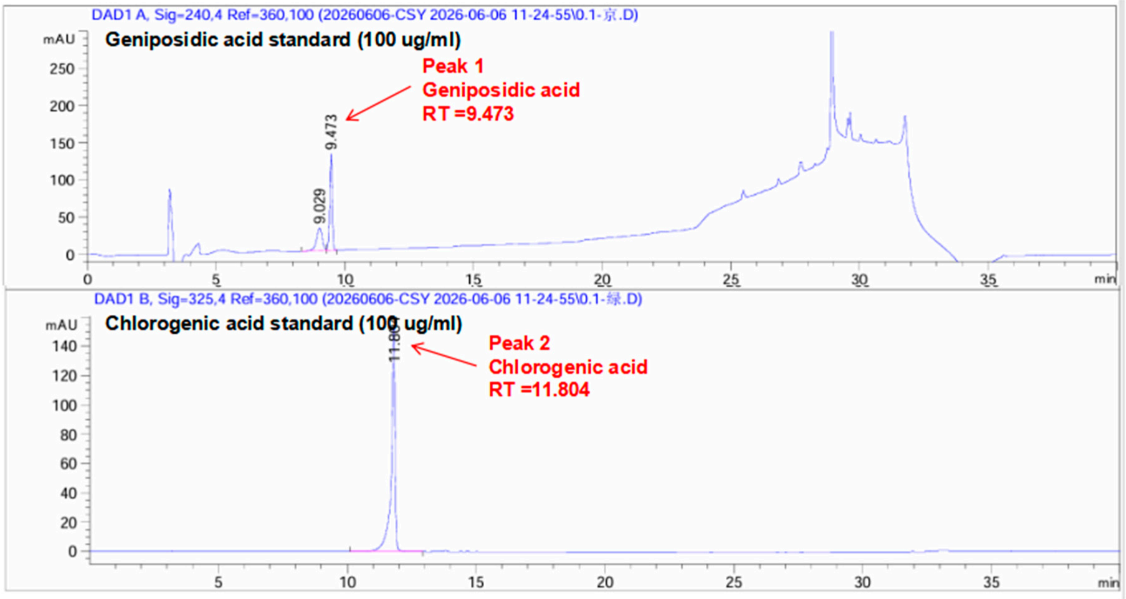

Figure S2. HPLC-DAD chromatograms of the mixed reference standards containing geniposidic acid and chlorogenic acid (100 μg/mL each).

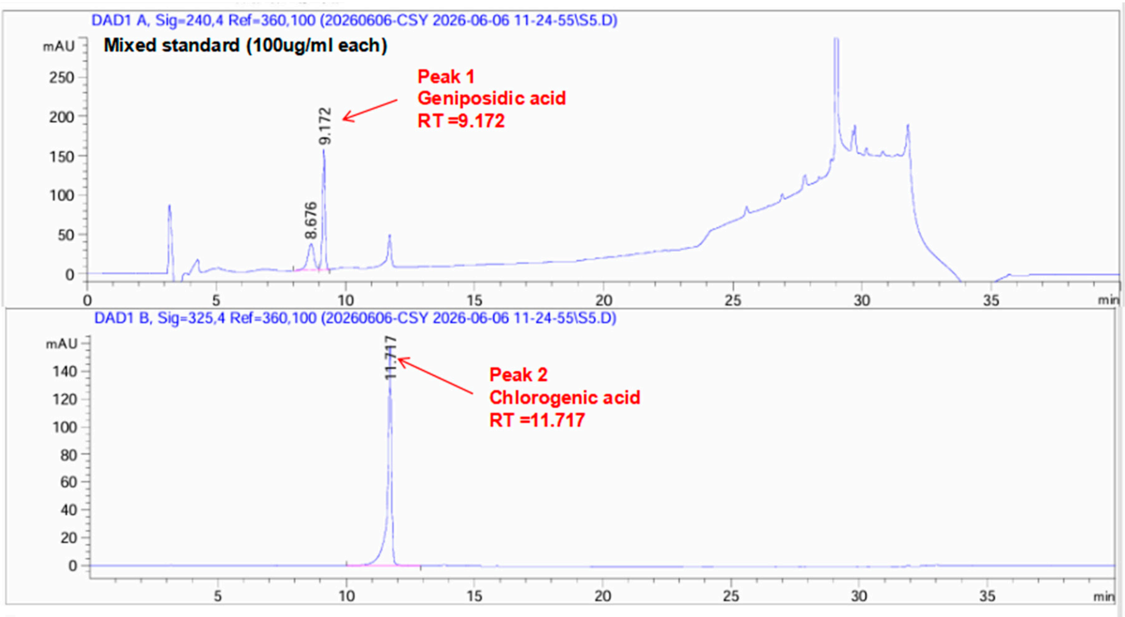

Figure S3. HPLC-DAD chromatograms of EUE showing the geniposidic acid peak at 240 nm in replicate analyses.

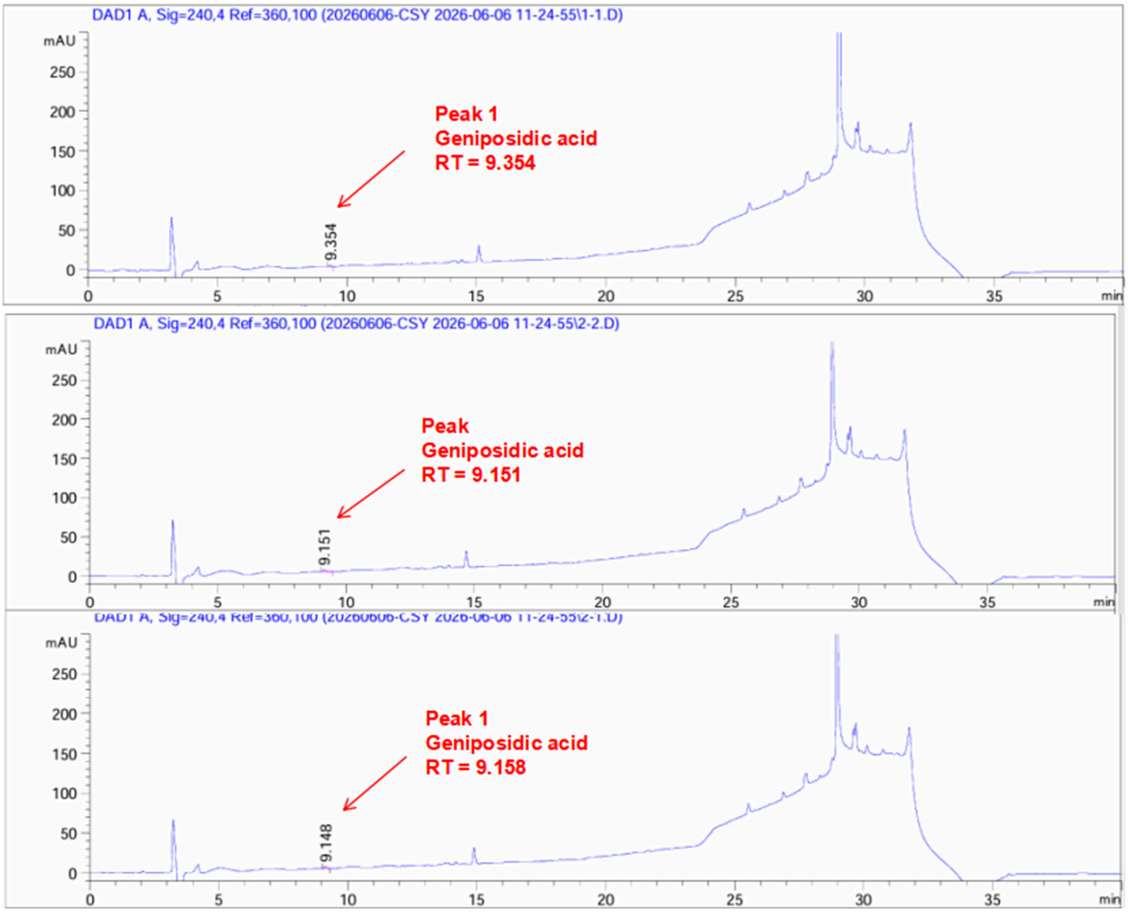

Figure S4. HPLC-DAD chromatograms of EUE showing the chlorogenic acid peak at 325 nm in replicate analyses.

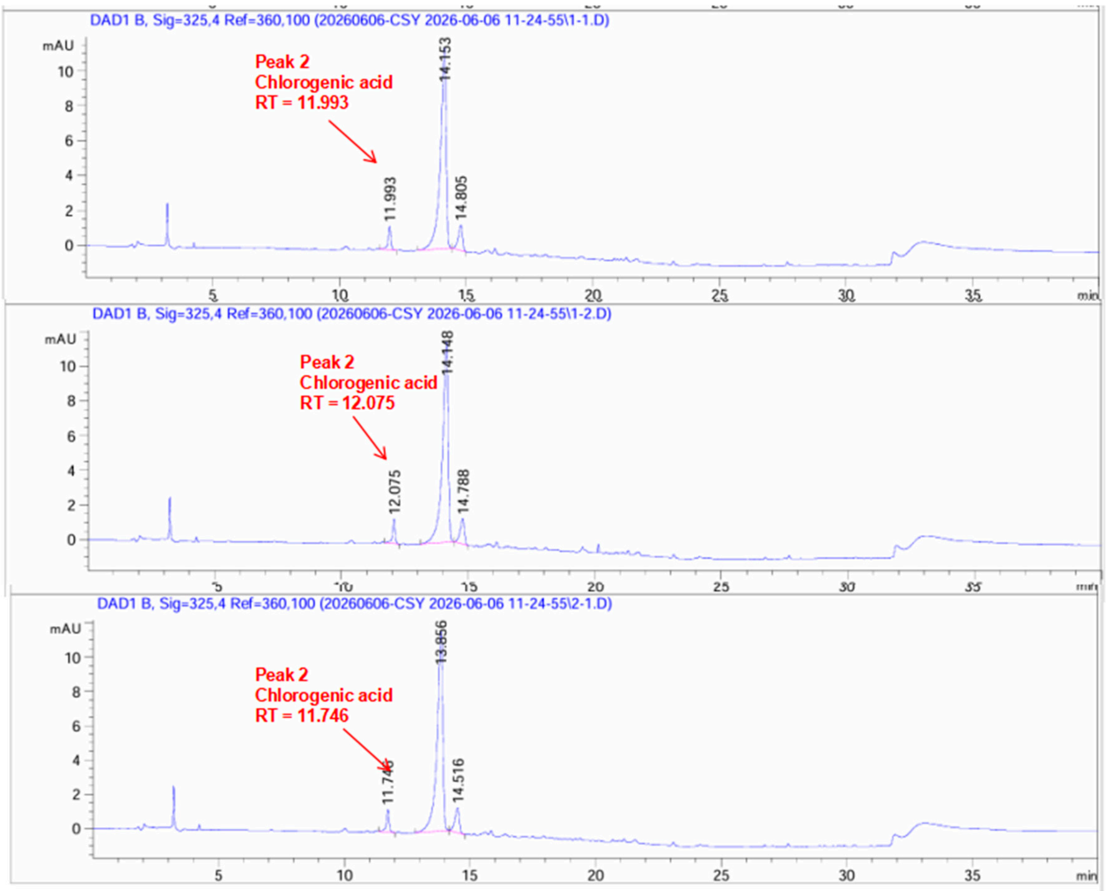
DAD1 B, Sig=325,4 Ref=360,100 (20260606-CSY 2026-06-06 11-24-55\1-1.D)
mAU
Peak 2
Chlorogenic acid
RT = 11.993
11.993
14.153
14.805
min
DAD1 B, Sig=325,4 Ref=360,100 (20260606-CSY 2026-06-06 11-24-55\1-2.D)
mAU
Peak 2
Chlorogenic acid
RT = 12.075
12.075
14.148
14.788
min
DAD1 B, Sig=325,4 Ref=360,100 (20260606-CSY 2026-06-06 11-24-55\2-1.D)
mAU
Peak 2
Chlorogenic acid
RT = 11.746
13.856
14.516
min

Figure S5. Overlay chromatograms of reference standards and EUE. (A) Geniposidic acid single standard versus mixed standards. (B) Chlorogenic acid single standard versus mixed standards. (C) EUE versus mixed standards at 325 nm for chlorogenic acid. (D) EUE versus mixed standards at 240 nm for geniposidic acid.

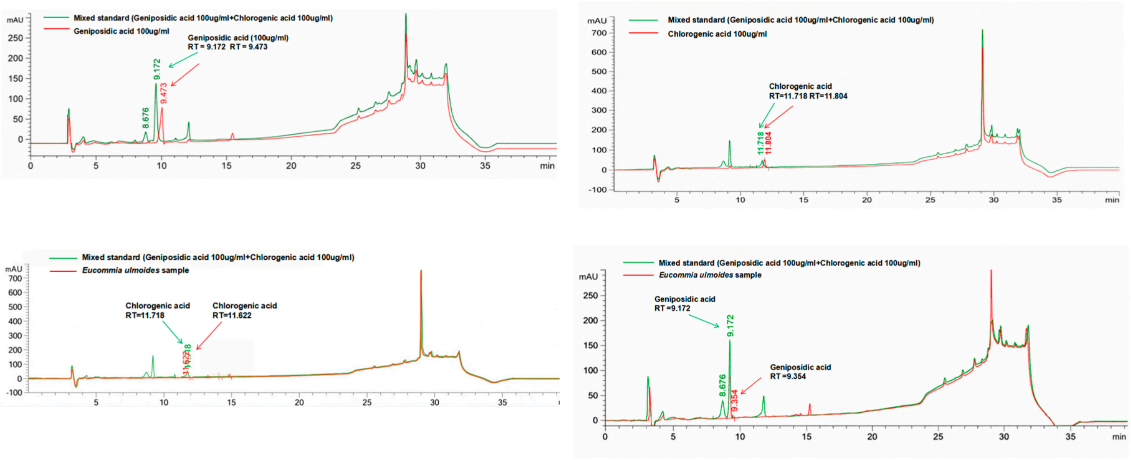

Online DAD spectral characterization

Figure S6. Online DAD UV absorption spectra of the representative marker compounds. (A) Geniposidic acid. (B) Chlorogenic acid.

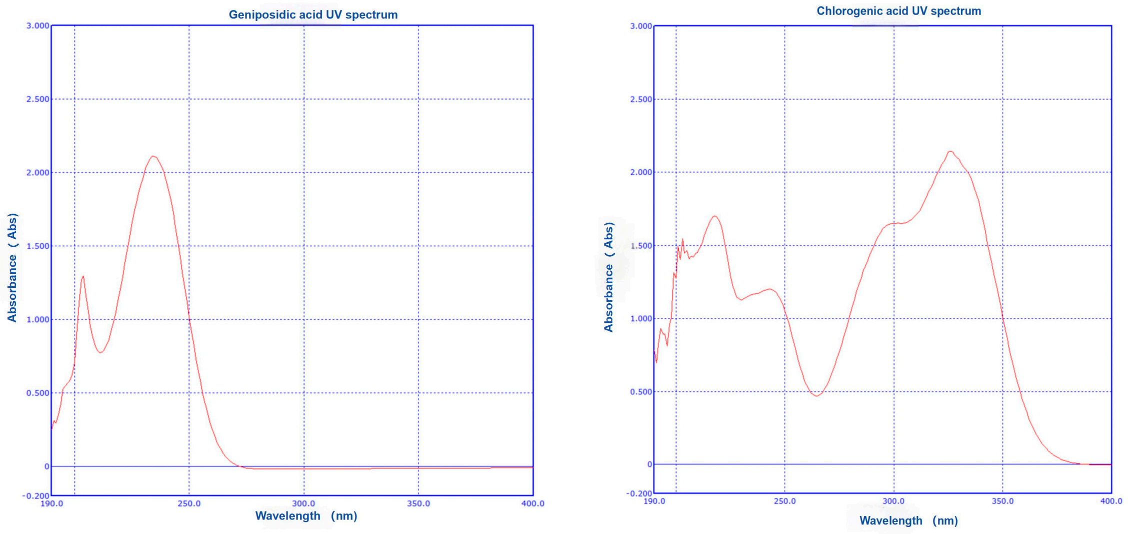

Calibration curves and content determination

Figure S7. Calibration curves of chlorogenic acid and geniposidic acid used for marker quantification.

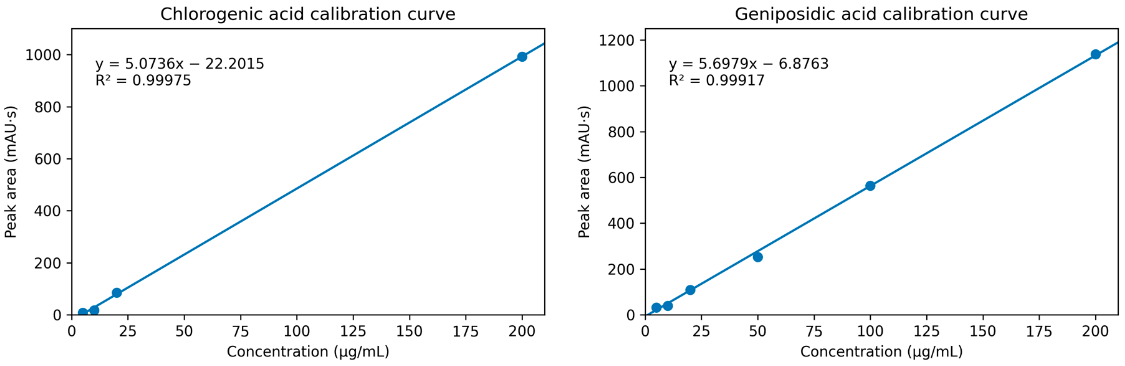

Table S3. Calibration curves and quantitative results for marker compounds in EUE.

**Compound**	**Regression equation**	**R^2**	**Concentration in sample solution**	**Content in EUE**
Chlorogenic acid	y = 5.0736x − 22.2015	0.99975	6.42 ± 0.02 µg/mL	3.21 ± 0.01 mg/g extract
Geniposidic acid	y = 5.6979x − 6.8763	0.99917	3.76 ± 0.30 µg/mL	1.88 ± 0.15 mg/g extract

Interpretation. Together, these chromatographic and online DAD spectral data provide marker-based chemical characterization of the EUE used in the present study.
